# Frequent but asymmetric niche shifts in *Bulbophyllum* orchids support environmental and climatic instability in Madagascar over Quaternary time scales

**DOI:** 10.1186/s12862-016-0586-3

**Published:** 2016-01-19

**Authors:** Alexander Gamisch, Gunter Alexander Fischer, Hans Peter Comes

**Affiliations:** Department of Ecology and Evolution, University of Salzburg, A-5020 Salzburg, Austria; Kadoorie Farm and Botanic Garden Corporation, Lam Kam Road, Tai Po, NT, Hong Kong SAR, China

**Keywords:** Macroecological niche, Niche shift vs. niche conservatism, Ancestral character reconstruction, Orchidaceae

## Abstract

**Background:**

Species or clades may retain or shift their environmental niche space over evolutionary time. Understanding these processes offers insights into the environmental processes fuelling lineage diversification and might also provide information on past range dynamics of ecosystems. However, little is known about the relative contributions of niche conservatism versus niche divergence to species diversification in the tropics. Here, we examined broad-scale patterns of niche evolution within a Pliocene–Pleistocene clade of epiphytic *Bulbophyllum* orchids (30 spp.) whose collective distribution covers the northwest and eastern forest ecosystems of Madagascar.

**Results:**

Using species occurrence data, ecological niche models, and multivariate analyses of contributing variables, we identified a three-state niche distribution character for the entire clade, coinciding with three major forest biomes *viz*. phytogeographical provinces in Madagascar: *A*, Northwest ‘Sambirano’; *B*, ‘Eastern Lowlands’; and *C*, ‘Central Highlands’. A time-calibrated phylogeny and Bayesian models of niche evolution were then used to detect general trends in the direction of niche change over the clade’s history (≤5.3 Ma). We found highest transitions rates between lowlands (*A* and *B*) and (mostly from *B*) into the highland (*C*), with extremely low rates out of the latter. Lowland-to-highland transitions occurred frequently during the Quaternary, suggesting that climate-induced vegetational shifts promoted niche transitions and ecological speciation at this time.

**Conclusions:**

Our results reveal that niche transitions occurred frequently and asymmetrically within this Madagascan orchid clade, and in particular over Quaternary time scales. Intrinsic features germane to *Bulbophyllum* (e.g., high dispersal ability, drought tolerance, multiple photosynthetic pathways) as well as extrinsic factors (ecological, historical) likely interacted to generate the niche transition patterns observed. In sum, our results support the emerging idea of dramatic environmental and climatic fluctuations in Madagascar during the recent geological past, which overturns the long-held paradigm of long-term stability in tropical forest settings. The generality of the patterns and timings reported here awaits the availability of additional comparative studies in other Madagascan endemics.

**Electronic supplementary material:**

The online version of this article (doi:10.1186/s12862-016-0586-3) contains supplementary material, which is available to authorized users.

## Background

Whether species or clades retain or shift their environmental niche space over time is a key question relevant to our understanding of speciation and large-scale patterns of diversification [1–7]. At the species level, the ability of populations to colonize and spread into new (e.g., climatic) niches supports a role for ecological speciation in which divergent natural selection on ecologically important traits promotes population isolation through adaptation to new environments [1, 8–10]. By contrast, niche conservatism is the inability of populations to adapt to new environmental conditions and but nonetheless may facilitate allopatric speciation by maintaining ancestral niches and niche-related traits over time [1, 2, 7]. Moreover, at the phylogenetic level, geographical patterns of niche shifts versus niche conservatism can offer insights into the environmental processes fuelling lineage diversification and may also provide important information on the range dynamics of species and ecosystems over geological time (e.g., [7, 9, 11–13]). However, few phylogenetic-dating studies have explicitly addressed this latter issue in tropical biota, and those that did, mainly focussed on animals (e.g., [13, 14] but see [15–17] for plant examples).

Madagascar is one of the world’s richest biodiversity hotspots [18], with about 3.2 % and 2.8 % of the estimated global total for number of species of vascular plants and vertebrates, respectively [19, 20]. This rich tropical species diversity is commonly attributed to the island’s long-term geological isolation from Africa (<150 million years ago, Ma) and India (<90 Ma), a high geological and topographical complexity, and regionally pronounced and locally steep environmental (e.g., climatic, elevational) gradients [21–23]. Originally, the forest ecosystems and climates of Madagascar, like elsewhere in the tropics, were believed to have been relatively stable through most of their Cenozoic history, thus allowing the built-up of high species diversity (e.g., [24–26]; but see [7, 27–29]). However, Madagascar’s pollen and vertebrate subfossil record is scarce (e.g., [30, 31]), which makes it difficult to formulate *a priori* hypotheses about the impact of past vegetation-climate changes on the niche evolution of local biota. Nonetheless, in agreement with the few palaeo-data available (e.g., [32–35]), there is growing evidence from phylogeographical studies of forest-dwelling plants and animals (e.g., [36–38]) that dramatic vegetational shifts took place in Madagascar during the climatic fluctuations of the Quaternary (≤2.6 Ma). Furthermore, using palaeoclimatic modelling, Rakotoarinivo et al. [29] have shown that Quaternary changes in vegetation-precipitation relationships in Madagascar exerted a strong influence on plant species richness, as exemplified by palms (Arecaceae) of the island’s eastern rainforest biomes. It is feasible, therefore, that Madagascan forest ecosystems underwent repeated range expansion/contraction cycles due to Quaternary palaeoclimatic change, which in turn promoted phylogenetic niche shifts along steep environmental gradients [21–23, 39–42]. An alternative, mutually non-exclusive hypothesis is that niche conservatism had likewise a role in facilitating speciation and lineage divergence through the maintenance of long-term allopatry in proposed glacial refuge locations [21], notably habitat isolation caused by aridification of low-elevation/montane river catchments [23, 29].

Molecular phylogenetic tools are increasingly used to examine patterns and processes of diversification in plant and animal groups from Madagascar [23, 38, 43–47]. However, to our knowledge, no study to date has specifically examined niche evolution within a Madagascan clade using phylogenetic reconstruction and ecological modelling as integrative tools of the emergent field of ‘phyloclimatic analysis’ (e.g., [11, 13, 48] and references therein; but see [47]). For example, if niche shifts play an important role in phylogenetic history, we would expect that species niches have non-overlapping geographical distributions and are phylogenetically over-dispersed (i.e., mixed among clades); conversely, under phylogenetic niche conservatism, species sharing the same niche will be phylogenetically clustered [1, 4, 5, 10, 47, 49, 50]. Another prediction of the niche shift hypothesis is that the distribution of niche variables across a phylogenetic tree is not significantly different from a random distribution, while under niche conservatism one would expect a strong tendency of related species to resemble each other in such variables more than if taken at random from the tree [3, 15, 51, 52].

**Fig. 1:**
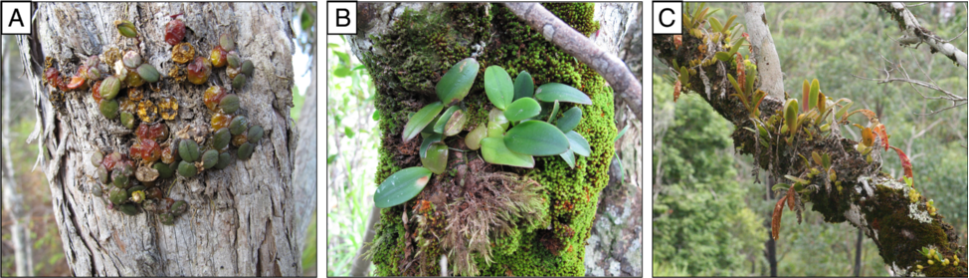
Members of Madagascan *Bulbophyllum* clade C in their different forest habitats. (**a**) *B. complanatum* from the seasonally dry region of Northwest Madagascar, (**b**) *B. elliotii* from the humid rainforest of the eastern coast, (**c**) *B. occultum* from the mid-to-high elevation humid rainforest of the eastern slopes. Photos by G. A. Fischer

Here, we adopted a phyloclimatic analysis approach to determine broad-scale patterns of niche evolution during the diversification of a well-defined clade of the pantropic genus *Bulbophyllum*Thouars (Orchidaceae, Epidendroideae) from Madagascar and adjacent islands. With about 2200 to 2400 described species, *Bulbophyllum* is one of the largest genera within the orchid family, and has a major center of diversity in Madagascar (*>*210 spp.), where species mainly occur as epiphytes in a wide range of rainforest habitats [53–56]. Based on molecular phylogenetic evidence [56–59], Madagascan *Bulbophyllum* forms a monophyletic group of Late Miocene age (*c*. 10.5 Ma) with two major lineages: a species-rich core clade (‘A–B’) mainly distributed in eastern rainforest of mid-to-high elevation (*c*. 800–1300 m), and a species-poor clade (‘C’) that is eco-geographically more varied (Fig. 1, see below). That said, new species of *Bulbophyllum* continue to be discovered in Madagascar and the Mascarenes ([60, 61]; G. A. Fischer, unpubl. data) but are rare and threatened with extinction, as typical for other rainforest taxa of these islands (e.g., [29]).

In this study, we used a time-calibrated species-level phylogeny of Madagascan *Bulbophyllum* ‘clade C’ (30 spp.), previously constructed from plastid and nuclear markers [59], as a framework to examine patterns and processes of niche evolution during the clade’s Plio-/Pleistocene diversification (*c.* ≤ 5.3 Ma). In general, we used a broad-scale, macroecological perspective at the clade-level rather than looking at the niche space of individual species, as the majority of *Bulbophyllum* species have small to very small ranges, so that sample sizes would be too low to implement such an approach for most species (e.g., [62, 63]). Accordingly, based on georeferenced occurrence data and bioclimatic/elevation variables of the entire clade, we first identified and characterized the major partitions of the dataset in terms of their (supposedly) realized niche space (sensu Soberón [64, 65] using, respectively, fuzzy clustering/ordination analyses in conjunction with ecological niche models (ENMs) [66] and predicted niche occupancy (PNO) profiles [11]. Second, we traced transitions between these realized niches across the tree and used a Bayesian modelling framework to test for a general trend in the direction of niche change through time. Finally, we assessed the extent of phylogenetic signal in variables of the clade’s niches. Together, our results reveal that niche transitions occurred frequently and asymmetrically within this Madagascan orchid clade, and in particular over Quaternary time scales. This supports the emerging idea that climate-induced vegetational shifts in the recent geological past gave rise to current eco-geographical patterns of biota in this tropical biodiversity hotspot.

## Methods

### Study system and phylogenetic framework

Taxonomically, Madagascan *Bulbophyllum* ‘clade C’ comprises members of sects. *Bifalcula, Humblotiorchis,* and *Calamaria* with altogether 32 recognized species ([56]; G. A. Fischer, B. Gravendeel, J. Hermans, A. Sieder, M. Kiehn, J. Andriantiana, P. J. Cribb, unpubl. data; see (Table 1). These leaf-succulent epiphytes (or rarely lithophytes) are mainly restricted to Northwest/East Madagascar (27 spp.) and/or adjacent islands (Mascarenes: La Réunion/Mauritius, Comores, Seychelles; 3 spp.), with two found in Madagascar and/or East Africa (*B. humblotii*, *B. malawiense*; see [59]). Preferential habitats range from seasonally dry to coastal/littoral and humid evergreen forests at various altitudes (0–1800 m) ([56, 58, 67]; G. A. Fischer, B. Gravendeel, J. Hermans, A. Sieder, M. Kiehn, J. Andriantiana, P. J. Cribb, unpubl. data). As typical for *Bulbophyllum*, most clade C species are adapted to insect (usually fly) mediated outcrossing (e.g., [68, 69]), excepting eight species (indicated in Table 1) that are polymorphic for both outcrossing and selfing variants [70, 71]. This intra-specific mating type polymorphism, however, is not correlated with the macroecological niche states and their contributing variables reported herein (A. Gamisch, unpubl. data), and thus will not be considered further.

**Table 1:** Niche-cluster membership of 604 data point localities itemized per species of *Bulbophyllum* clade C (sects. *Bifalcula*, *Calamaria*, *Humblotiorchis*), and outgroups, used as tip-states for the reconstruction of ancestral niche states *A, B,* and *C* (see Fig. 4). Note, two clade C species are not included in the present study (i.e., *B. cryptostachium* Schltr., *B. sp. nov.* ‘*F*’)

		Niche states		Coding
Section/species	A	B	C	
*Bifalcula*				
*B. capuronii* Bosser	0	4	0	B
*B. complanatum* ^*a*^ H.Perrier	11	0	0	A
*B. implexum* Jum. & H.Perrier	12	0	0	A
*B. minutum* Thouars	0	12	0	B
*B. sp. nov. A*	0	3	0	B
*B. sp. nov. B*	0	0	1	C
*Calamaria*				
*B. bicoloratum* ^*a*^ Schltr.	3	10	43	C
*B. cirrhoglossum* H.Perrier	0	4	0	B
*B. elliotii* Rolfe	1	19	0	B
*B. erectum* ^*a*^ Thouars	21	48	0	AB
*B. hildebrandtii* Rchb.f.	17	0	14	AC
*B. histrionicum* G.A.Fischer & P.J.Cribb	20	0	0	A
*B. incurvum* Thouars	0	0	5	C
*B. lecouflei* Bosser	2	3	0	AB
*B. luteobracteatum* Jum. & H.Perrier	0	0	4	C
*B. malawiense* B.Morris	0	0	2	C
*B. obtusatum* ^*a*^ (Jum. & H.Perrier) Schltr.	1	0	9	C
*B. occultum* ^*a*^ Thouars	1	61	86	BC
*B. pervillei* Rolfe	0	18	0	B
*B. pusillum* ^*a*^ (H.Perrier) G.A.Fischer & P.J.Cribb	2	2	35	C
*B. quadrifarium* ^*a*^ Rolfe	0	11	1	B
*B. rubrum* Jum. & H.Perrier	9	0	0	A
*B. ruginosum* H.Perrier	0	0	3	C
*B. senghasii* G.A.Fischer & A.Sieder	1	0	0	A
*B. sp. nov. C*	0	7	0	B
*B. sp. nov. D*	0	0	1	C
*B. sp. nov. E1*	12	0	0	A
*B. sp. nov. E2*	1	0	0	A
*B. trifarium* Rolfe	8	0	2	A
*Humblotiorchis*				
*B. humblotii* ^*a*^ Rolfe	4	35	6	B
*Alcistachys*				
*B. variegatum* Thouars	0	12	3	B
*Inversiflorum*				
*B. cardiobulbum* Bosser	0	0	10	C
*Kainochilus*				
*B. horizontale* Bosser	0	0	4	C

^a^Clade C species polymorphic for (outcrossing/selfing) mating type [71]

The present study capitalizes on a nearly complete molecular phylogeny of clade C (comprised of 30 spp. plus one outgroup species each of related sects. *Alcistachys, Kainochilus*, and *Inversiflorum*; Table 1) as previously reconstructed from an eight-gene dataset of five plastid and three nuclear markers [59]. Phylogenetic analyses, using maximum parsimony (MP) (Paup* v. 4.0b10, [72]) and Bayesian inference (BI) (Mrbayes v. 3.1.2, [73]), were conducted on the combined plastid/nuclear dataset (6505 bp in total). Bayesian posterior probability (PP > 0.95) and parsimony bootstrap percentage (BP ≥ 85) were used as estimates of strong clade support. The trees retrieved identified sects. *Bifalcula* (6 spp.) and *Humblotiorchis* (1 sp.) as successive sister taxa to sect. *Calamaria* (23 spp.). An estimate of absolute age for each node was obtained from a relaxed molecular clock analysis in Beast v. 1.6.1 [74] using multiple fossils and geological events for calibration (for details see [59]). The resulting maximum clade credibility (MCC) chronogram is used here to represent the clade C phylogeny and to trace the history of niche shifts across this phylogeny (see below).

### Identification and characterization of macroecological niches

The fundamental niche space of species or higher taxa can be separated into variables of biotic interactions and resource-consumer dynamics (‘Eltonian niche’) or into variables of broad-scale environmental conditions (‘Grinnellian niche’) [64]. The interplay between ‘Eltonian’ processes (e.g., competitive exclusion), ‘Grinnellian’ factors (e.g., climate) and others (e.g., dispersal limitation, demographic constraints), defines the realized niche [64]. For the purpose of this study we rely on a reasonable approximation of the realized niche based on climatic and elevation variables of a comprehensive locality dataset for all 30 clade C species (plus outgroups) [65, 75]. This database was assembled from two main sources: (1) own field data gathered during expeditions in Madagascar (G.A.F. and A. Sieder, Vienna) and La Réunion (A.G.); and (2) voucher specimen data (from the period 1850–2010) obtained from herbaria (G, K, MO, NEU, P, TAN, W, WU). Specimen localities without coordinates were geotagged using an internal Gazetteer of Kew Royal Botanic Gardens, topographical maps of Madagascar (1:100.000), and GoogleEarth™. We discarded duplicate presence records per species as well as implausible locality data (e.g., outside a 1 km radius of the Gazetteer locality and/or those of likely misidentified species accessions). In total, the final dataset comprised 604 georeferenced point localities (coordinates available on request), with the number per species ranging from 1 to 148 (mean ± SD, 18.84 ± 28.71).

For each locality in the database, we extracted altitude and 19 bioclimatic (‘bioclim’) data layers for current conditions (~1950–2000) at 30 arc-seconds resolution from the WorldClim database v. 1.4 ([76]; http://www.worldclim.org), using the R-package Raster v. 1.9-70 [77]. To investigate how these 604 data points group in multi-dimensional environmental space into a specific number of different clusters, we used fuzzy *C*-means (FCM) clustering [78–81]. Compared to conventional (‘hard’) clustering methods, FCM clustering has the advantage that it allows gradual memberships to clusters measured as degrees (0, 1) [82]. This ‘degree of belonging (or fuzziness)’ is defined by a membership coefficient (*m*), which quantifies a data point’s membership to all the given clusters. FCM clustering is usually regarded more ‘natural’ than ‘hard’ clustering, especially when based on continuous (e.g., environmental, climatic) variables as used here [79, 81, 82].

The 604 data points were fuzzy clustered on the basis of their Euclidian distances calculated from the environmental variables (altitude, bioclim 1–19) using the *vegdist* function of the R package Vegan v. 2.0-3 [83]. The resultant distance matrix was multi-dimensionally scaled using the *cmdscale* command in R (i.e., the matrix was scaled such that the distances between the data points were approximately equal to their Euclidian distance). We employed seven different fuzzy validity indices (detailed in [Additional file 1]) to determine the number of clusters existing in the dataset using the functions *cmeans* and *fclustIndex* of the R package e1071 v. 1.6 [84]. The number of clusters (*K*) was set to vary from 2 to 15, each combined with a fuzziness scheme at ten steps (*m* = 1.1, 1.2 … 2.0), resulting in a total of 140 combinations. Each *cmeans* run converged before the maximum of 200 iterations was reached. Overall, this FCM analysis identified three clusters (hereafter termed ‘*A*’, ‘*B*’, and ‘*C*’) to which samples were assigned according to their maximum *m* value (see Results).

We compared the environmental space between the three FCM clusters using principal component analysis (PCA) in R (*prcomp* command). The PCA was performed on the matrix of variables (altitude, bioclim 1–19) and the component scores of each data point were projected in two dimensions. For each cluster pair, we assessed significance between the first two components (PC1, PC2) using Mann–Whitney U tests. This non-parametric approach was used because PC1 did not fulfil the parametric assumption of normal distribution based on Kolmogorov-Smirnov tests (data not shown).

ENMs were built independently for each FCM cluster using Maxent v. 3.3.3e [66], a maximum entropy method frequently employed for species distribution modelling (SDM) (e.g., [85, 86]). Based on the locality data assigned to each cluster, ENMs were developed using the 19 bioclim data layers plus altitude (see above) to visualize the geographic space and overlap of these clusters, and to compare their ranges with known phytogeographical zones in Madagascar. For each cluster, model performance was assessed using 100 bootstrap replicates (70 % training, 30 % testing). The area under the ‘Receiver Operating Characteristic (ROC) Curve’ (AUC; [87, 88]) was averaged across replicates to determine if models should be removed (AUC < 0.7). Maxent outputs of continuous probabilities for habitat suitability were converted to presence/absence predictions using a logistic threshold maximizing the absolute value of training sensitivity plus specificity (e.g., [89]).

Using the approach of Evans et al. [11], we further characterized each FCM cluster by predicted niche occupancy (PNO) profiles for particular environmental variables using the R package Phyloclim v. 0.8.1 [90]. As input, this program uses Maxent raw data (i.e., cumulative probabilities of habitat suitability) for creating ‘unit area histograms of suitability’, which illustrate the predicted occupancy of an environmental variable by a given set of locality data (in this case of clusters *A, B,* and *C*). As predictive variables we selected six that received relatively high loadings in the PCA (see Results).

### Ancestral niche reconstructions and testing alternative models of niche transition

We used the submodule Multistate of BayesTraits v. 1.0 ([91]; http://www.evolution.rdg.ac.uk/BayesTraits.html) to reconstruct ancestral niche states under a continuous-time Markov model of discrete character evolution. For each clade C (and outgroup) species, we coded niche state according to the majority of localities per species falling within a certain FCM cluster (hereafter referred to as ‘niche’), and as’polymorphic‘if at least one third of those localities fell into a different niche (see Table 1, showing near-equal species frequencies across niches: *A*: 9.5, *B*: 11.5, *C*: 12). Probabilities of transitions were calculated together with the probabilities of niche states for all internal nodes of the last 5000 post-burn-in trees from the BI analysis of clade C (with outgroup taxa pruned prior to analysis; [58]), which thus accounts for uncertainty in the phylogeny’s branch lengths and topology. The Markov chain Monte Carlo (MCMC) analyses were run for 5,050,000 generations, with a *ratedev* parameter of 250 (obtained from initial test runs), a reversible-jump hyperprior with an exponential prior (mean seeded from a uniform distribution on the interval 0 to 30), and a burn-in of 50,000 generations. The reconstructed ancestral niche states were plotted onto the Beast-derived (MCC) chronogram of clade C. Phylogenetic niche shifts were quantified by comparing the most probable states between ancestral and descendant nodes [16, 17, 92].

We used a modelling framework within BayesTraits to estimate niche transition rates (*q*) and to detect any general trend in the direction of niche change (see Table 2). Specifically, we compared a full (six-parameter) model that allowed transitions to vary freely to (1) each of six models with zero uni-directional transitions in all pairwise combinations of states (i.e., *q*_AB_ = 0; *q*_AC_ = 0; … *q*_CB_ = 0); and (2) one model with zero bi-directional transitions from *C* (i.e., *q*_CB_ = 0, *q*_CA_ = 0). Note, this latter model was guided by our ancestral niche reconstructions (see Results). We used Tracer v. 1.5 [93] to compute the marginal likelihoods of the full and constrained models. For model comparison, we used the Bayes Factor (BF) ‘test statistic’ of 2 log(marginal likelihood [unconstrained model]) – log(marginal likelihood [constrained model]) [93–95]. Evidence against the constrained model (i.e., the null hypothesis, H_0_) was considered to be ‘positive’ (BF value = 2–6), ‘strong’ (6–10), or ‘very strong’ (>10).

**Table 2:** Results of testing alternative models of niche transition in BayesTraits using logarithmic Bayes Factor (BF) comparisons

Model	Marginal likelihood	Standard error	2(log BF)	*q*_AB_	*q*_AC_	*q*_BA_	*q*_BC_	*q*_CA_	*q*_CB_
No constraint	−34.74	+/− 0.017	-	77.03	68.17	68.99	71.50	41.74	53.28
*q*_AB_ = 0	−37.73	+/− 0.020	5.93	0	57.61	64.94	79.81	38.02	63.57
*q*_AC_ = 0	−36.64	+/− 0.019	3.53	74.17	0	67.35	81.99	31.09	53.85
*q*_BA_ = 0	−37.37	+/− 0.020	5.12	75.50	76.98	0	70.84	63.44	59.58
*q*_BC_ = 0	−38.20	+/− 0.023	5.85	78.44	83.30	74.54	0	42.40	42.62
*q*_CA_ = 0	−34.38	+/− 0.017	−0.75	75.47	62.09	72.48	71.12	0	51.80
*q*_CB_ = 0	−35.21	+/− 0.017	0.80	80.33	65.13	69.27	66.25	40.37	0
*q*_CB_ = 0, q_CA_ = 0	−35.00	+/− 0.017	0.46	77.64	59.20	70.45	63.67	0	0

### Phylogenetic signal in environmental variables

To provide a complementary perspective on the clade’s niche evolution, we used Blomberg’s *K* [51] to test for correlations between environmental variables and phylogenetic relatedness. In general, this test statistic is used to assess phylogenetic signal by asking whether a given phylogeny (topology and branch lengths) better fits a set of quantitative tip data than expected for data randomly permuted across the tips of the tree [15, 51, 96]. Values of *K* range from zero to infinity, where *K* < 1 indicates weak phylogenetic signal, and *K* > 1 strong signal, implying that close relatives are, respectively, more or less divergent for traits than expected under a Brownian model (*K* = 1; [51]). First, based on the locality data, we calculated the centroid value of each principal component (PC1, PC2) per FCM cluster, and also the median values of environmental variables (altitude, bioclim 1–19) for each clade C species. Those ‘trait’ values were then used as input data of the R package Picante v. 1.5-2 [97] for calculating values of *K* for each trait over 100 trees from the BI analysis (derived from [59]). Significance of *K* was assessed by comparing observed patterns of the variance of independent contrasts of the trait to a null model by shuffling taxa across the tips of the phylogeny (999 randomized data sets).

## Results

### Macroecological niche identification and characterization

Our results of the FCM cluster analysis offered more than one optimal solution for partitioning the 604 occurrence data of Madagascan *Bulbophyllum* clade C (plus outgroups) in environmental space (Additional file 1). In detail, of the seven validity measures employed, two (i.e., Dunn’s separation and Xie-Beni indices) suggested clustering of the samples into three groups (*K* = 3, with *m* = 1.1 or 2.0), while two others (Bezdek’s partition coefficient and Bezdek’s partition entropy) favoured *K* = 4 with *m* = 1.1. By contrast, the remaining indices suggested *K* = 12, 13, or 14 as best partitions (bold and underlined in [Additional file 1]). However, we favoured *K* = 3 (with *m* = 1.1) as suggested by the majority of validity indices (four out of seven) when considering their next to optimal values (bold in [Additional file 1]). Accordingly, the 604 locality data were assigned to three clusters depending on the maximum *m* value per sample (i.e., *A*: 125, *B*: 249, *C*: 229).

**Fig. 2:**
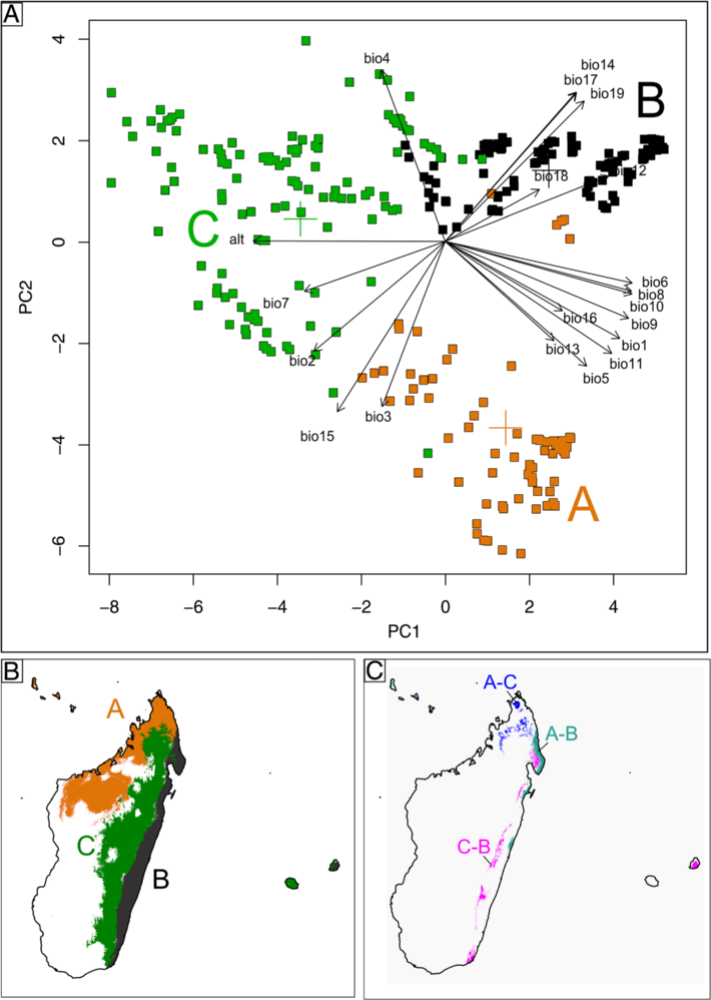
Macroecological clusters *viz*. niches (*A, B, C*) of *Bulbophyllum* clade C from Madagascar and adjacent islands (Comores, La Réunion, Mauritius). **a** Principal component analysis (PCA) biplot of 604 occurrence data (squared symbols) of clade C species (plus three outgroup species) based on 19 bioclimatic variables (bio 1–19) and altitude (alt). Each data point is coloured according to its hard cluster membership as defined by fuzzy *C*-means (FCM) clustering. Coloured crosses indicate centroids. (Note: the factor loadings do not correspond to the x or y axes, see [Additional file 2] for factor loadings). **b** Binary ecological niche models (ENMs) for clusters *A*, *B*, and *C* using a maximum training sensitivity plus specificity logistic threshold. **c** Range overlap between the ENMs of clusters (*A*–*B*, *A*–*C*, *C*–*B*) based on the same threshold

Figure 2a shows the two-dimensional PCA plot of the 604 samples as assigned to their respective FCM clusters (i.e., *A, B,* or *C*). Samples of clusters *C* and *B* mainly differed along the first component (PC1, accounting for 52 % of the total variance), which was positively correlated mainly with altitude, measures of temperature (e.g., bio6, bio10), and precipitation (bio12) (Additional file 2). By contrast, samples of *A* and *B* were largely separated along PC2 (27 %), which was mainly positively correlated with temperature seasonality (bio4) but negatively with both precipitation seasonality (bio15) and isothermality (bio15); (Additional file 2). As a consequence, clusters *A* and *C* differed along both PC axes. However, all pairwise comparisons between clusters revealed significant differences for each of the two axes tested (Mann–Whitney U tests, all *P* < 0.001).

### Spatial-climatic niche characterisation

**Fig. 3:**
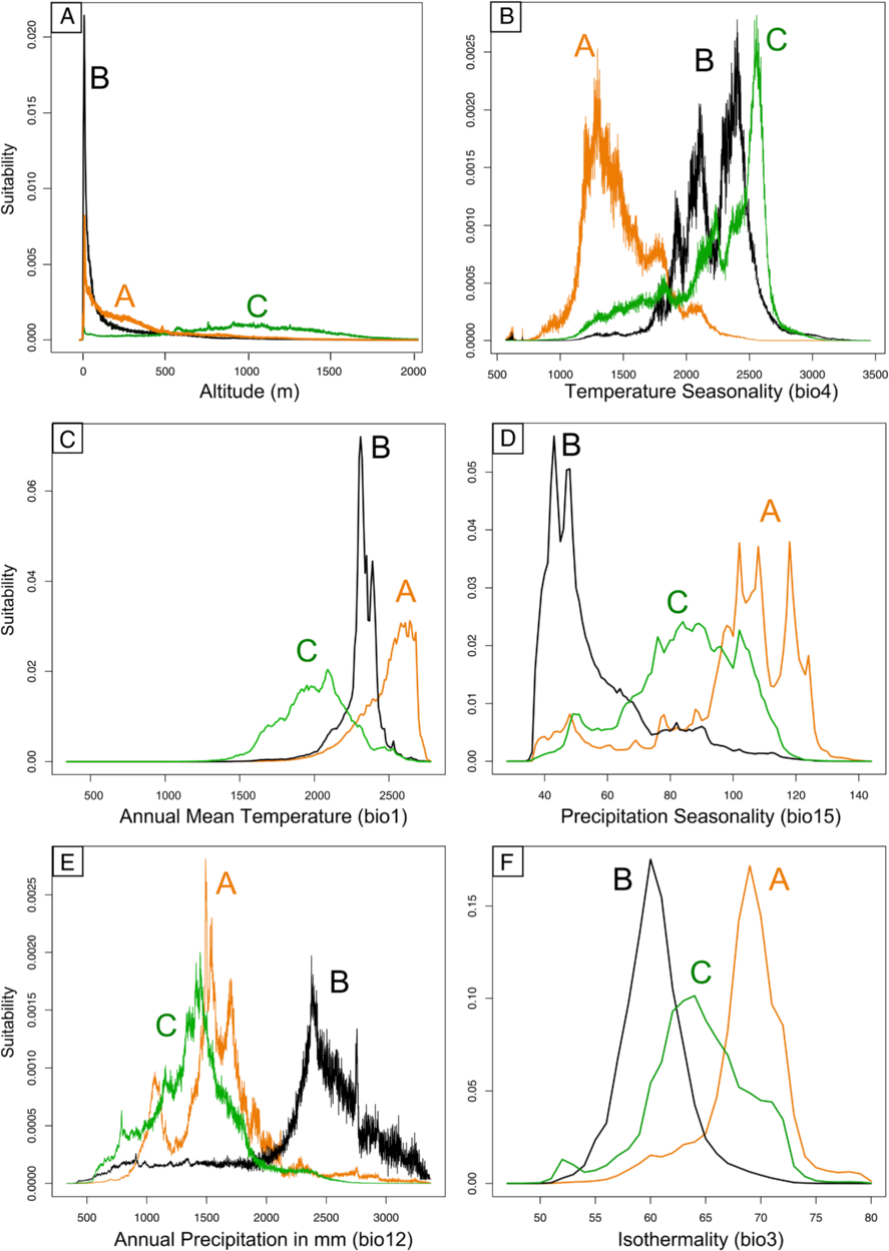
Predicted niche occupancy (PNO) profiles of niche clusters *A, B,* and *C* of Madagascan *Bulbophyllum* clade C (see Fig. 2): for (**a**) altitude, (**b**) temperature seasonality (bio4), (**c**) annual mean temperature (bio1), (**d**) precipitation seasonality (bio15), (**e**) annual precipitation (bio12), and (**f**) isothermality (bio3)

The ENMs generated for clusters *A, B,* and *C* had high AUC values (range: 0.978–0.992), indicating good predictive performance. Based on these models, the predicted distributions of these clusters in Madagascar (Fig. 2b) are largely parapatric with little spatial overlap (*c.* 1.6–4.6 %; Fig. 2c). Considering further the PNO profiles of each cluster for selected environmental variables (Fig. 3), as well as phytogeographic provinces in Madagascar [20, 98–101], the predicted distributions of clusters *A, B,* and *C* (hereafter ‘niches’) can be characterized as follows. Niche *A* occupies the seasonally dry region of Northwest Madagascar at low-to-mid elevations (0–800 m) (Fig. 2b), and is characterized mainly by constantly high temperatures, coupled with low annual precipitation that markedly varies across seasons (Fig. 3). Phytogeographically, niche *A* largely coincides with the ‘Sambirano’ rainforest domain [20, 98], which receives heavy seasonal rainfall due to the influence of both easterly trade winds and Indian monsoon currents, but also extends into the western seasonally dry forest at about the same elevation [20, 98–101]. By contrast, niche *B* coincides with the ‘Eastern Lowlands’ (Fig. 2b) of coastal (littoral) forest, intersected by marshland, and humid rainforest at low-to-mid elevation (*c.* 0–800 m). When compared to niches *A* and *C*, niche *B* is intermediate in both annual temperature and temperature seasonality; however, it receives the highest annual precipitation, which is continually supplied throughout the year (Fig. 3). Finally, niche *C* largely coincides with the eastern slopes and plateau of the ‘Central Highlands’ (800–1200 m) (Fig. 2b) characterized by, respectively, mid-to-high elevation humid rainforest and sclerophyllous forest/mountain (‘Philippia’) scrubland [100]. In terms of annual temperature regime, niche *C* is the coldest and most variable one; it receives similar levels of annual precipitation as niche *A* but at a more constant rate throughout the year (Fig. 3).

### Ancestral niche reconstructions and patterns of niche transition

**Fig. 4:**
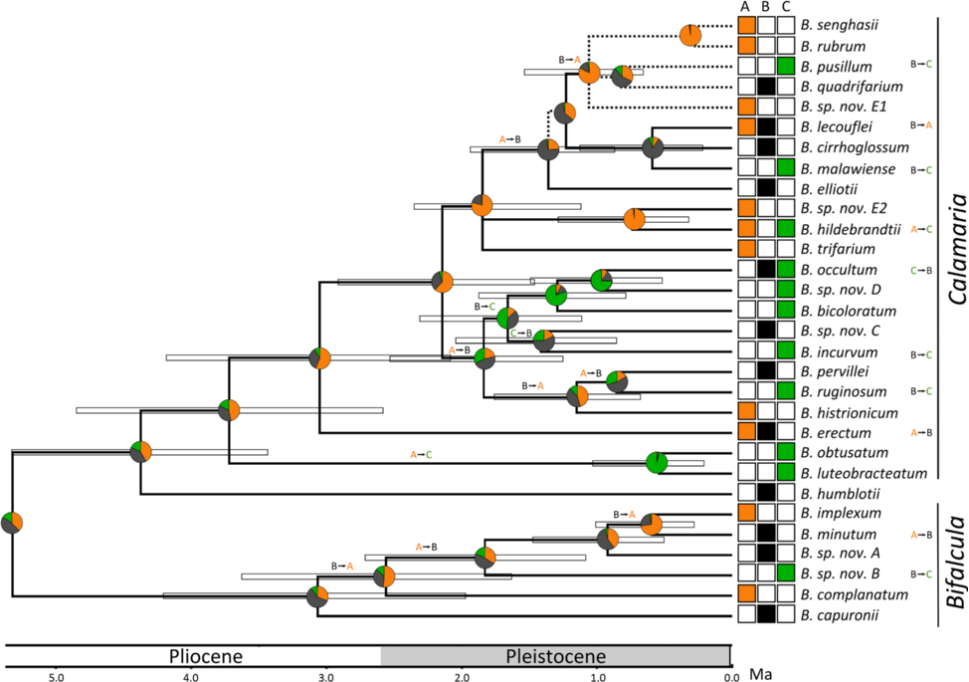
Niche shifts of Madagascan *Bulbophyllum* clade C. BayesTraits MCMC reconstruction of ancestral niche states (*A, B, C*) plotted onto a Beast-derived chronogram of Madagascan *Bulbophyllum* clade C with branch lengths proportional to time (million of years ago, Ma; modified from [59]). Boxes at tips correspond to the character state coding (see Table 1), while pie charts at nodes represent probabilities for ancestral states. Niche shifts and their directionality are indicated at internal branches or next to taxon names if associated with tip branches. Stippled lines indicate phylogenetic posterior probability (PP) support below 0.95. Horizontal bars indicate 95 % highest posterior density (HPD) for node ages

The Bayesian MCMC reconstruction of ancestral niche states (*A, B, C*) along the chronogram of clade C (Fig. 4) placed its crown node (*c.* 5.3 Ma) in either *A* or *B* with near equal probabilities. This analysis further revealed frequent niche transitions within this clade, especially in more recent geological times. If we consider only transitions associated with well-supported sister species or subclades (>0.95 PP), then niche shifts may have occurred up to 21 times independently, and all but one of those fall into the Quaternary (≤2.6 Ma; Fig. 4). Altogether 11 transitions appear on internal nodes, including eight from *A* to *B* or vice versa, but only one each from *A* and *B* to *C*, and one from *C* to *B*. The 10 remaining transitions are located on terminal tip branches (associated with speciation events), including six (mostly from *B* to *C*)*,* three from *A* to *B* or vice versa, and only one from *C* to *B*. Clearly, these inferences about the number of independent transitions and reversals have to be treated with some caution because ancestral states at several nodes appeared equivocal (Fig. 4). Nonetheless, these results qualitatively suggest frequent niche transitions between *A* and *B* and almost irreversible transitions from mainly *B* to *C*.

**Fig. 5:**
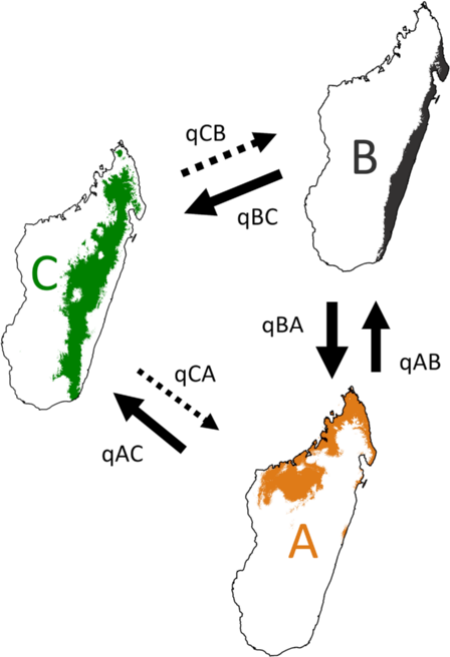
Transition probabilities between niche states (*A, B, C*). The size of the arrows is proportional to the pairwise transition rates (*q*_AB_, *q*_BA_, *q*_AC_, *q*_CA_, *q*_CB_, *q*_BC_) estimated by BayesTraits, whereas the dashed lines indicate transition rates not significantly different from zero (see Table 2)

Estimates of transition rates in BayesTraits (Table 2) confirmed this partly asymmetrical niche change within clade C. According to the unconstrained (full) model, transitions were most probable between niches *A* and *B*, followed by those from either *B* to *C* or from *A* to *C*, whereas reversals from *C* were rendered highly unlikely (see also Fig. 5). Note that this analysis assigned high probability to *A* to *C* transitions, despite being observed only two times along the phylogeny (Fig. 4; see above). When we compared the fit of the full model against each of the seven models with asymmetrical transitions (Table 2), there was ‘positive’ to ‘strong’ evidence against all four models with zero (uni-directional) transitions from *A* or *B* (BF = 3.53–5.93). However, none of the remaining models, constraining either uni- or bi-directional transitions from *C*, provided a worse fit than the full model (BF = −0.75–0.80) (Table 2).

### Phylogenetic signal

We found no phylogenetic signal of Blomberg’s *K* for the two principal components (PC1, PC2) and their constituent variables (altitude, bioclim 1–19) (all *K* < 1; Table 3). Moreover, for PC2 and all variables, the observed distribution of values across the tips of the phylogeny was not significantly different from a random distribution in most of the 100 BI trees analyzed (average 97 %; median *P* = 0.098–0.749). By contrast, for PC1, the percentage of trees rejecting the null hypothesis of a random signal was relatively high (84 %; median *P* = 0.03), indicating an even lesser amount of phylogenetic clustering than would be expected by chance (i.e., under a Brownian model of evolution).

**Table 3:** Tests for phylogenetic signal of Blomberg’s *K* for the centroid values of principal environmental components (PC1, PC2) per cluster (*A, B, C*), and the median values of contributing variables (altitude, bioclim 1–19) per species, over 100 Bayesian trees of *Bulbophyllum* clade C (derived from [59])

Variable	Median K	Median *P*	% of trees not rejecting H_0_ (*P* > 0.05)^a^
PC1	0.443	0.030	16
PC2	0.328	0.295	100
Altitude	0.354	0.297	99
Bio1 = Annual Mean Temperature	0.366	0.145	93
Bio2 = Mean Diurnal Range	0.399	0.098	93
Bio3 = Isothermality	0.395	0.101	93
Bio4 = Temperature Seasonality	0.319	0.340	100
Bio5 = Max Temperature of Warmest Month	0.383	0.121	87
Bio6 = Min Temperature of Coldest Month	0.355	0.218	96
Bio7 = Temperature Annual Range	0.371	0.120	87
Bio8 = Mean Temperature of Wettest Quarter	0.366	0.220	99
Bio9 = Mean Temperature of Driest Quarter	0.353	0.230	98
Bio10 = Mean Temperature of Warmest Quarter	0.377	0.170	97
Bio11 = Mean Temperature of Coldest Quarter	0.363	0.154	92
Bio12 = Annual Precipitation	0.420	0.198	100
Bio13 = Precipitation of Wettest Month	0.343	0.231	100
Bio14 = Precipitation of Driest Month	0.331	0.370	100
Bio15 = Precipitation Seasonality	0.331	0.319	100
Bio16 = Precipitation of Wettest Quarter	0.364	0.236	99
Bio17 = Precipitation of Driest Quarter	0.335	0.372	100
Bio18 = Precipitation of Warmest Quarter	0.277	0.749	100
Bio19 = Precipitation of Coldest Quarter	0.338	0.305	100

^a^Percentages of trees not rejecting the null hypothesis of random signal for given variable

## Discussion

Although tropical environments comprise numerous biodiversity hotspots [18], it is precisely in such environments where least is known about the patterns and processes that drive lineage diversification and niche evolution (e.g., [23, 102]). This is the first study to integrate time-calibrated phylogeny, ancestral niche reconstructions, and hypothesis testing of niche transitions to explore the relative importance of niche conservatism versus niche divergence in the evolutionary history of a plant lineage from the tropical biodiversity hotspot of Madagascar. Based on the evidence from *Bulbophyllum* orchids, our results reveal an exceptionally high number of broad-scale macroecological niche shifts during the Plio-Pleistocene diversification of ‘clade C’ (30 spp.) in general, and over Quaternary time scales, in particular. Furthermore, this pattern is accentuated by partly asymmetrical niche transitions with little evidence for reversals from highland to lowland areas. Although there are limitations to the methods used (e.g., niche classification based on macroenvironmental conditions only [47] and at the clade level rather than for individual species; equivocal character states at several nodes of the phylogeny), our broad-scale patterns and estimates provide novel insights that can be used for more explicit hypothesis testing to understand historical and ecological factors driving plant diversification in Madagascar.

### Niche differentiation and lability within Madagascan *Bulbophyllum* clade C

Although all clade C species occur in tropical forest biomes, our FCM clustering in conjunction with the PCA (Fig. 2a) and our ENM results (Fig. 2b) revealed the presence of three macroecological niches (*A*, Northwest ‘Sambirano’; *B*, ‘Eastern Lowlands’; and *C*, ‘Central Highlands’), with ‘parapatric’ but *de facto* non-overlapping ranges (Fig. 2c) thereby largely coinciding with major provinces for the phytogeography of Madagascar [20, 98–101]. Interestingly, 26 of the 30 species included in this study can be assigned unequivocally to one of these three niches (Table 1, Fig. 4), suggesting that (broad-scale) niche conservatism prevails at the intra-specific level. Conversely, those species featuring multiple niche states (i.e., *B. erectum*, *B. hildebrandtii, B. lecouflei*, *B. occultum*) should be ideal candidates for fine-scale studies of incipient ecological speciation.

Our ancestral state reconstructions (Fig. 4) revealed significant evolutionary lability of niches (*A, B, C*) within clade C. Nonetheless, in several cases, niche conservatism has possibly been important in facilitating species divergence through the maintenance of long-term allopatry (e.g., regarding the sister-species *B. occultum*/*B. bicoloratum*; A. Gamisch and U. Jaros, unpubl. data; see also Fig. 4). However, across the entire clade, bioclimatic niche conservatism outweighs respective niche shifts by a ratio of only 2:1, which is considerably lower than the ratio previously reported (25:1) for vascular plants at a global scale [5]. In fact, the total number of 21 transitions inferred for clade C is exceptionally high when compared to other, similar-sized plant lineages that have been identified as undergoing bioclimatic niche shifts over Late Tertiary/Quaternary time scales. As for two examples, six biome shifts were reconstructed within a clade of African *Coccinia* [16], and just only two within a clade of South American *Leucocoryne* [103]. Clearly, these inferences about the number of independent transitions and reversals in *Bulbophyllum* clade C have to be treated with some caution due to the ambiguity in ancestral character reconstruction. In general, this difficulty to estimate ancestral states with confidence can have many different causes (e.g., taxonomic misclassification, incomplete taxon sampling, phylogenetic uncertainty, inappropriate state categorization, high transition rates among states; [104–106]). In the present case, we suspect this uncertainty is most likely caused by the high rates of transition among niche states ([107–109], M. Pagel, pers. comm.), as also inferred from the results of our BayesTraits analyses (Fig. 5, Table 2; see below). However, the statistical nature of this inference needs to be explored in more detail using simulations.

In view of the high number of niche shifts within Madagascan *Bulbophyllum* clade C, it is not unexpected that we found no phylogenetic signal of Blomberg’s *K* for principal environmental components (PC1, PC2) and all their contributing variables (Table 3). Such lack of phylogenetic signal in ecological traits is generally interpreted as being indicative of high rates of niche evolution (*viz*. niche-related trait lability) among closely related species that rapidly diversified to fill new and/or empty niches [51, 110]. This also accords with altogether ten niche transitions associated with tip branches of the clade C phylogeny (Fig. 4), suggesting that niche shifts had a significant role in recent species diversification (see below).

But what factors then could have promoted these frequent niche transitions? One possible explanation is that these tropical orchids possess a suite of characteristics that make them better suited for such transitions than other plant groups. For instance, *Bulbophyllum* species, like other orchids, possess dust-like and wind dispersed seeds [111–113]. This high dispersal ability may facilitate both habitat selection and stabilizing selection on phenotypic (e.g., niche-related) traits [52, 114, 115]. Additionally, *Bulbophyllum* produces pseudobulbs with water-storage tissue, which indicates tolerance to drought stress [67, 116, 117]. Notably, too, this genus is highly variable in photosynthetic assimilation pathways, with species exhibiting C_3_, CAM (Crassulacean Acid Metabolism), and/or C_3_-CAM intermediate pathways [118–120]. Although little is known about this physiological variation in Madagascan *Bulbophyllum* (for which only C_3_ photosynthesis has been reported so far [121]), CAM is generally believed to assist species in occupying a wider range of habitats, including both semiarid and (sub) tropical environments [117, 120, 122, 123]. Together, these general characteristics may help explain the evolutionary versatility of clade C species to exploit new environmental niches and adapt to novel selective regimes (e.g., [124]). Although the species-specific (intrinsic) traits involved remain unknown, they likely interacted in a complex way with both ecological (abiotic and/or biotic) as well as historical (extrinsic) factors to generate the remarkable pattern of niche shifts exhibited by this group (see below).

### Directionality of niche transitions

Given the high number of niche shifts and the lack of a phylogenetic signal for environmental variables within *Bulbophyllum* clade C, one may conjecture that niches varied at random along the phylogeny [7, 16]. This, however, was not observed as we detected several trends in the direction, frequency, and timing of particular niche shifts in this orchid group. Both ancestral niche reconstructions (Fig. 4) and BayesTraits analyses (Fig. 5, Table 2) concurred in that niche shifts within clade C mainly took place from niche *A* to *B*, and in the opposite direction, with either niche or, as observed, predominantly *B* acting as ancestral source for transitions into *C*. Remarkably, however, reversals back from the dryer and colder high-elevation niche *C* to the warmer lowlands (*A* or *B*) were hardly observed (Fig. 4), and also deemed improbable by the hypothesis testing framework (Fig. 5, Table 2).

The PCA results (Fig. 2a) offer some tentative explanation for the above transition patterns in suggesting differential constraints along the major environmental trajectories (PC1, PC2) linking the three niches. Accordingly, shifts between the lowland niches *A* and *B* would need to overcome constraints only along PC2, which may have required adaptive responses to differences in seasonality (temperature, precipitation) and isothermality. By contrast, shifts from *B* to *C* mainly proceed along PC1, implying adaptations to overall temperature regime, precipitation, as well as higher elevation. Finally, shifts between *A* and *C* would require adaptations along both trajectories (PC1, PC2), and likely therefore are the most severely constrained. In fact, transitions from *A* to *C* are rarely observed across the phylogeny (Fig. 4), despite being rendered highly probable by BayesTraits (Fig. 5, Table 2).

However, apart from these different ecological constraints, historical factors may have had a role in shaping the direction and frequency of niche transitions. For instance, Madagascan pollen fossil records, even though scarce, suggest that the primary forest vegetation of the ‘Central Highlands’ was more widespread at lower elevation during the colder (glacial) periods of the Late Quaternary, while receding towards the upper slopes of mountains during warmer inter-/postglacials [32–35]. More recently, this ‘Quaternary vegetational shift’ hypothesis has gained support from population genetic studies of forest-dependent rodent species from northern Madagascar [37, 38]. For *Bulbophyllum*, there is likewise preliminary evidence from species-specific ENMs for the Last Glacial Maximum (LGM; *c.* 21,000 year BP) that suitable habitat of high-elevation forest-dwellers (e.g., *B. occultum*) expanded downwards at that time (A. Gamisch, unpubl. data). It is feasible, therefore, that during the Quaternary climate-induced vegetational shifts (in both elevation and latitude) promoted niche shifts in *Bulbophyllum* not only between lowlands (*A* and *B*) but also from lowland (*B*) to highland (*C*) areas. In addition, our results show that 90 % of all speciation events within clade C (27/30) fall into the Quaternary, and when associated with niche shifts, they mostly occur from *B* to *C* (five out of ten; Fig. 4). This trend in the timing and frequency of low-to-highland transitions further supports the hypothesis that Quaternary vegetational shifts had an influential role not only in promoting niche shifts *per se* but also in driving ecological speciation in Madagascan *Bulbophyllum*.

An outstanding question is why reversals back to lowland niches, i.e., from *C* to *A* or *B*, were hardly observed (Fig. 4) and modelled with zero probability (Table 2). Given the high frequency of *B* to *C* transitions, and lesser abiotic (i.e., climatic) constraints along this trajectory relative to those from *A* to *C* (see above), one would have expected *C* to *B* reversals to occur at least to some extent. Of course, this asymmetry might simply reflect insufficient time elapsed for relevant physiological traits to (re-)evolve in response to warmer and wetter lowland (*B*) conditions. On the other hand, we may exclude the possibility that reversals are limited by higher extinction rates in niche *C*, because the time-calibrated phylogeny of the entire clade (Fig. 4) best fits a constant-rates model of diversification without extinction [59]. In other words, niche *C* is probably not a mere biogeographical ‘sink’ where extinction exceeds colonization. However, with recourse to the ‘Quaternary vegetational shift’ hypothesis, a viable historical scenario is that, associated with inter-/postglacial change to a warmer climate, the upward retreat of the lower limits of the Central Highland forest vegetation severed habitat connections to the Eastern Lowland forest [32, 35, 38]. In the case of *Bulbophyllum*, this hypothesis implies that, in addition to climate and topography, historical factors likewise imposed constraints on niche shifts from *C* to *B*. However, it is also possible that any asymmetry in niche shifts in this plant group from highland to lowland forests may have more to do with biotic interactions (e.g., competition with well-adapted residents; lack of suitable pollinators) than it does with physiological adaptation to lowland climates and/or the fragmentation of past migration routes (see also [125]).

Clearly, a full understanding why clade C species exhibit frequent yet irreversible transitions to higher elevation habitats (niche *C*) over geologically recent (Quaternary) time scales would require fine-scaled analyses. This, for instance, may involve sister-species or species populations distributed on either side of the *B*–*C* niche boundary (e.g., *B. pervillei/B. ruginosum*), and including genetic, phenotypic, ecological, and distributional (ENM) data for present and past (LGM) climate conditions to test for asymmetric migration and dispersal rates. It also needs to be explored in more ecological detail which biotic factors may have a major role in disconnecting the fundamental and realized niches of these tropical orchids, especially due to the role of competitors and/or pollinators. Nevertheless, the present results concur with phylogenetic reconstructions in other plant and animal taxa from tropical and extra-tropical regions, showing a general trend of niche transitions from lower to higher elevation habitats (e.g., [52, 86, 126–130]), whereas well-supported cases of reverse shifts seem to be rare (but see [39]). Whether this reflects actual patterns or bias in data collection remains unclear, and is worth addressing in greater depth (see [131] for the reminiscent case of mainland-island transitions).

## Conclusions

On this broad macroecological scale, our findings highlight an unusually high incidence of niche transitions in an epiphytic orchid lineage from Madagascar, which seems to overturn the long-held paradigm of long-term stability in tropical forest ecosystems [24–26]; but see [7, 27–29]. Over the entire Plio-/Pleistocene history of *Bulbophyllum* clade C, the highest transitions rates were between the two lowland rainforest niches, Northwest ‘Sambirano’ (*A*) and ‘Eastern Lowlands’ (*B*), and into the mixed-forest/scrubland niche of the ‘Central Highlands’ (*C*), with extremely low rates out of the latter. Intrinsic features germane to this orchid group (e.g., high dispersal ability, drought tolerance, multiple photosynthetic pathways) as well as extrinsic factors (ecological, historical) likely interacted to generate this partly asymmetrical niche transition pattern. Our results also tend to suggest that Quaternary vegetational shifts had an important role in promoting niche transitions in general, and shaping recent speciation via Eastern Lowland-to-Central Highland shifts in particular. The generality of these patterns and timings awaits the availability of additional comparative studies in other Madagascan plant endemics, which might also shed light on the Central Highland biome as a possible ‘end of the colonization road’. Finally, this work clearly demonstrates the need for species-level sampling and molecular ecological analyses of *Bulbophyllum* clade C to better understand the relative importance of historical and contemporary factors driving speciation in this orchid group.

### Availability of supporting data

The data sets supporting the results of this article are included within the article (and its Additional files 1 and 2). The phylogenetic data sets [59] supporting the results of this article are available in the Dryad Digital Repository [doi:10.5061/dryad.35935].

## Additional files

Additional file 1:
**Fuzzy C-means (FCM) clustering analysis of 604 locality data points of** ***Bulbophyllum*** **clade C (and outgroup taxa).** (DOC 209 kb) Additional file 2:
**Rankings of loadings for the first two principal components (PC1, PC2) of 604** ***Bulbophyllum*** **clade C (plus outgroup) locality values of 20 environmental variables (altitude, bioclim 1–19).** (DOC 40 kb)
