# Supplementary material for: Frequent but asymmetric niche shifts in Bulbophyllum orchids support environmental and climatic instability in Madagascar over Quaternary time scales
Source: BMC Evol Biol. 2016 Jan 19;16:14. doi: 10.1186/s12862-016-0586-3 (PMC4717530; doi:10.1186/s12862-016-0586-3)
Supplement: Additional file 1: — Fuzzy C-means (FCM) clustering analysis of 604 locality data points of Bulbophyllum clade C (and outgroup taxa). (DOC 209 kb) [file 12862_2016_586_MOESM1_ESM.doc]

**Additional file 1**

**Gamisch et al. “Frequent but asymmetric niche shifts in *Bulbophyllum* orchids support environmental and climatic instability in Madagascar over Quaternary time scales”**

**Table S1** Fuzzy *C*-means (FCM) clustering analysis of 604 locality data points of *Bulbophyllum* clade C (and outgroup taxa) performed on their Euclidian distance matrix based on 20 environmental variables (altitude, bioclim 1–19). Seven fuzzy validity indices were used to determine the optimal number of clusters (*K*) and degree of fuzziness (*m*) based on 140 binary combinations of set values (*K* = 2–15, *m* = 1.1, 1.2 … 2.0). For each index, values are highlighted as ‘optimal’ (bold, underlined) and ‘next to optimal’ (only bold).

| ***K*** | ***m*** | **Fuzzy validity index** | | | | | | |
| --- | --- | --- | --- | --- | --- | --- | --- | --- |
|  |  | **Fuzzy hyper volume** | **Average partition density** | **Xie-Beni index** | **Fukuyama-Sugeno index** | **Bezdek´s partition coefficient** | **Bezdek´s partition entropy** | **Dunn´s separation index** |
| 2 | 1.1 | 3.18E+05 | 6.17E-04 | 3.85E-04 | -2.26E+08 | 9.78E-01 | 3.70E-02 | 2.18E-02 |
| 2 | 1.2 | 3.25E+05 | 6.03E-04 | 3.78E-04 | -2.20E+08 | 9.48E-01 | 8.32E-02 | 5.47E-02 |
| 2 | 1.3 | 3.36E+05 | 6.28E-04 | 3.66E-04 | -2.17E+08 | 9.23E-01 | 1.24E-01 | 1.02E-02 |
| 2 | 1.4 | 3.51E+05 | 6.63E-04 | 3.51E-04 | -2.14E+08 | 9.00E-01 | 1.63E-01 | 3.40E-02 |
| 2 | 1.5 | 3.71E+05 | 6.47E-04 | 3.36E-04 | -2.11E+08 | 8.77E-01 | 2.01E-01 | 3.40E-02 |
| 2 | 1.6 | 3.94E+05 | 6.66E-04 | 3.21E-04 | -2.07E+08 | 8.54E-01 | 2.39E-01 | 3.40E-02 |
| 2 | 1.7 | 4.19E+05 | 6.50E-04 | 3.07E-04 | -2.03E+08 | 8.31E-01 | 2.75E-01 | 3.40E-02 |
| 2 | 1.8 | 4.45E+05 | 6.37E-04 | 2.93E-04 | -1.99E+08 | 8.09E-01 | 3.09E-01 | 1.46E-02 |
| 2 | 1.9 | 4.70E+05 | 6.02E-04 | 2.79E-04 | -1.94E+08 | 7.88E-01 | 3.41E-01 | 1.38E-02 |
| 2 | 2 | 4.94E+05 | 5.71E-04 | 2.66E-04 | -1.89E+08 | 7.67E-01 | 3.71E-01 | 1.38E-02 |
| 3 | 1.1 | 2.22E+05 | 8.59E-04 | 2.20E-04 | -3.40E+08 | **9.91E-01** | **1.75E-02** | **7.15E-02** |
| 3 | 1.2 | 2.32E+05 | 7.78E-04 | 2.23E-04 | -3.35E+08 | 9.70E-01 | 5.00E-02 | 3.41E-02 |
| 3 | 1.3 | 2.47E+05 | 7.79E-04 | 2.21E-04 | -3.32E+08 | 9.54E-01 | 8.36E-02 | 3.41E-02 |
| 3 | 1.4 | 2.72E+05 | 8.43E-04 | 2.17E-04 | -3.27E+08 | 9.32E-01 | 1.32E-01 | 3.41E-02 |
| 3 | 1.5 | 3.08E+05 | 8.06E-04 | 2.11E-04 | -3.19E+08 | 9.02E-01 | 1.94E-01 | 3.41E-02 |
| 3 | 1.6 | 3.53E+05 | 7.70E-04 | 2.04E-04 | -3.09E+08 | 8.66E-01 | 2.64E-01 | 3.44E-02 |
| 3 | 1.7 | 4.04E+05 | 7.06E-04 | 1.96E-04 | -2.96E+08 | 8.28E-01 | 3.35E-01 | 3.44E-02 |
| 3 | 1.8 | 4.58E+05 | 6.72E-04 | **1.87E-04** | -2.83E+08 | 7.88E-01 | 4.05E-01 | 1.76E-02 |
| 3 | 1.9 | 5.13E+05 | 6.17E-04 | 1.78E-04 | -2.70E+08 | 7.50E-01 | 4.71E-01 | 1.76E-02 |
| 3 | 2 | 5.67E+05 | 5.75E-04 | **1.69E-04** | -2.57E+08 | 7.14E-01 | 5.32E-01 | 1.76E-02 |
| 4 | 1.1 | 2.13E+05 | 1.02E-03 | 2.96E-04 | -3.68E+08 | **9.94E-01** | **1.14E-02** | 2.75E-02 |
| 4 | 1.2 | 2.33E+05 | 1.09E-03 | 5.61E-04 | -3.63E+08 | 9.51E-01 | 8.44E-02 | 1.48E-02 |
| 4 | 1.3 | 2.52E+05 | 1.08E-03 | 5.54E-04 | -3.58E+08 | 9.28E-01 | 1.32E-01 | 1.76E-02 |
| 4 | 1.4 | 2.57E+05 | 9.67E-04 | 2.98E-04 | -3.46E+08 | 9.08E-01 | 1.68E-01 | 1.49E-02 |
| 4 | 1.5 | 2.95E+05 | 1.01E-03 | 2.89E-04 | -3.37E+08 | 8.75E-01 | 2.38E-01 | 1.24E-02 |
| 4 | 1.6 | 3.87E+05 | 8.38E-04 | 4.94E-04 | -3.29E+08 | 8.26E-01 | 3.47E-01 | 1.80E-02 |
| 4 | 1.7 | 4.10E+05 | 8.08E-04 | 2.54E-04 | -3.09E+08 | 7.99E-01 | 3.98E-01 | 2.12E-02 |
| 4 | 1.8 | 4.81E+05 | 7.47E-04 | 2.35E-04 | -2.92E+08 | 7.58E-01 | 4.81E-01 | 2.12E-02 |
| 4 | 1.9 | 5.55E+05 | 6.48E-04 | 2.16E-04 | -2.75E+08 | 7.17E-01 | 5.61E-01 | 1.24E-02 |
| 4 | 2 | 6.31E+05 | 5.67E-04 | 1.98E-04 | -2.58E+08 | 6.77E-01 | 6.37E-01 | 1.24E-02 |
| 5 | 1.1 | 2.05E+05 | 1.15E-03 | 3.29E-04 | -3.84E+08 | 9.83E-01 | 2.98E-02 | 3.19E-02 |
| 5 | 1.2 | 2.06E+05 | 1.10E-03 | 2.89E-04 | -3.87E+08 | 9.71E-01 | 5.50E-02 | 1.93E-02 |
| 5 | 1.3 | 2.21E+05 | 1.18E-03 | 2.86E-04 | -3.73E+08 | 9.33E-01 | 1.19E-01 | 3.40E-02 |
| 5 | 1.4 | 2.49E+05 | 1.30E-03 | 2.84E-04 | -3.59E+08 | 8.94E-01 | 1.90E-01 | 4.07E-02 |
| 5 | 1.5 | 2.85E+05 | 1.23E-03 | 2.72E-04 | -3.51E+08 | 8.67E-01 | 2.53E-01 | 1.88E-02 |
| 5 | 1.6 | 3.35E+05 | 1.08E-03 | 2.58E-04 | -3.42E+08 | 8.34E-01 | 3.29E-01 | 1.88E-02 |
| 5 | 1.7 | 4.00E+05 | 9.50E-04 | 2.42E-04 | -3.30E+08 | 7.97E-01 | 4.13E-01 | 1.88E-02 |
| 5 | 1.8 | 4.75E+05 | 8.58E-04 | 2.26E-04 | -3.16E+08 | 7.57E-01 | 5.01E-01 | 1.88E-02 |
| 5 | 1.9 | 5.58E+05 | 7.47E-04 | 2.09E-04 | -3.01E+08 | 7.16E-01 | 5.88E-01 | 1.88E-02 |
| 5 | 2 | 6.46E+05 | 6.38E-04 | 1.91E-04 | -2.86E+08 | 6.76E-01 | 6.72E-01 | 1.88E-02 |
| 6 | 1.1 | 1.93E+05 | 1.07E-03 | 3.16E-04 | -3.99E+08 | 9.89E-01 | 2.00E-02 | 4.00E-02 |
| 6 | 1.2 | 1.99E+05 | 1.15E-03 | 3.13E-04 | -3.91E+08 | 9.65E-01 | 6.13E-02 | 4.00E-02 |
| 6 | 1.3 | 2.22E+05 | 1.45E-03 | 3.96E-04 | -3.82E+08 | 9.19E-01 | 1.41E-01 | 3.31E-02 |
| 6 | 1.4 | 2.43E+05 | 1.25E-03 | 3.15E-04 | -3.72E+08 | 9.04E-01 | 1.83E-01 | 2.20E-02 |
| 6 | 1.5 | 2.91E+05 | 1.18E-03 | 3.12E-04 | -3.60E+08 | 8.67E-01 | 2.66E-01 | 4.00E-02 |
| 6 | 1.6 | 3.57E+05 | 1.01E-03 | 3.05E-04 | -3.46E+08 | 8.25E-01 | 3.62E-01 | 4.00E-02 |
| 6 | 1.7 | 4.53E+05 | 9.01E-04 | 2.76E-04 | -3.33E+08 | 7.79E-01 | 4.61E-01 | 1.88E-02 |
| 6 | 1.8 | 5.50E+05 | 8.21E-04 | 2.49E-04 | -3.17E+08 | 7.35E-01 | 5.61E-01 | 9.52E-03 |
| 6 | 1.9 | 6.33E+05 | 6.88E-04 | 2.70E-04 | -2.95E+08 | 6.86E-01 | 6.72E-01 | 3.27E-02 |
| 6 | 2 | 7.40E+05 | 5.81E-04 | 2.56E-04 | -2.77E+08 | 6.42E-01 | 7.68E-01 | 3.27E-02 |
| 7 | 1.1 | 1.86E+05 | 1.45E-03 | 4.89E-04 | -3.99E+08 | 9.81E-01 | 3.19E-02 | 2.02E-02 |
| 7 | 1.2 | 1.98E+05 | 1.22E-03 | 3.45E-04 | -4.03E+08 | 9.61E-01 | 7.04E-02 | 4.85E-02 |
| 7 | 1.3 | 2.09E+05 | 1.33E-03 | 3.13E-04 | -3.86E+08 | 9.30E-01 | 1.26E-01 | 4.00E-02 |
| 7 | 1.4 | 2.39E+05 | 1.34E-03 | 2.91E-04 | -3.76E+08 | 8.97E-01 | 1.94E-01 | 4.00E-02 |
| 7 | 1.5 | 3.09E+05 | 1.14E-03 | 5.90E-04 | -3.58E+08 | 8.41E-01 | 3.16E-01 | 3.25E-02 |
| 7 | 1.6 | 3.88E+05 | 9.71E-04 | 5.68E-04 | -3.43E+08 | 8.00E-01 | 4.14E-01 | 6.74E-03 |
| 7 | 1.7 | 4.88E+05 | 8.13E-04 | 5.64E-04 | -3.32E+08 | 7.54E-01 | 5.25E-01 | 2.65E-02 |
| 7 | 1.8 | 6.17E+05 | 7.50E-04 | 2.64E-04 | -3.08E+08 | 7.22E-01 | 6.16E-01 | 4.35E-02 |
| 7 | 1.9 | 7.24E+05 | 6.22E-04 | 3.93E-04 | -3.05E+08 | 6.77E-01 | 7.07E-01 | 1.61E-02 |
| 7 | 2 | 8.80E+05 | 5.33E-04 | 2.20E-04 | -2.67E+08 | 6.19E-01 | 8.43E-01 | 2.76E-02 |
| 8 | 1.1 | 1.55E+05 | 2.29E-03 | 2.79E-04 | -4.14E+08 | 9.89E-01 | 2.06E-02 | 4.00E-02 |
| 8 | 1.2 | 1.63E+05 | 2.15E-03 | 2.65E-04 | -4.04E+08 | 9.65E-01 | 5.92E-02 | 4.00E-02 |
| 8 | 1.3 | 1.99E+05 | 1.48E-03 | 5.47E-04 | -3.90E+08 | 9.25E-01 | 1.40E-01 | 8.33E-03 |
| 8 | 1.4 | 2.12E+05 | 1.50E-03 | 2.56E-04 | -3.85E+08 | 9.15E-01 | 1.65E-01 | 4.00E-02 |
| 8 | 1.5 | 3.28E+05 | 1.03E-03 | 5.69E-04 | -3.69E+08 | 8.38E-01 | 3.27E-01 | 2.40E-02 |
| 8 | 1.6 | 4.11E+05 | 1.01E-03 | 5.43E-04 | -3.50E+08 | 8.03E-01 | 4.18E-01 | 3.91E-02 |
| 8 | 1.7 | 4.81E+05 | 9.48E-04 | 3.16E-04 | -3.28E+08 | 7.58E-01 | 5.23E-01 | 2.63E-02 |
| 8 | 1.8 | 6.05E+05 | 7.87E-04 | 2.90E-04 | -3.09E+08 | 7.07E-01 | 6.45E-01 | 1.91E-02 |
| 8 | 1.9 | 7.32E+05 | 6.30E-04 | 4.17E-04 | -2.94E+08 | 6.73E-01 | 7.26E-01 | 2.66E-02 |
| 8 | 2 | 8.71E+05 | 5.32E-04 | 3.80E-04 | -2.75E+08 | 6.33E-01 | 8.26E-01 | 2.66E-02 |
| 9 | 1.1 | 1.74E+05 | 1.32E-03 | 5.89E-04 | -4.14E+08 | 9.84E-01 | 2.87E-02 | 2.19E-02 |
| 9 | 1.2 | 1.75E+05 | 2.14E-03 | 4.85E-04 | -4.16E+08 | 9.59E-01 | 7.13E-02 | 8.97E-03 |
| 9 | 1.3 | 1.86E+05 | 1.83E-03 | 6.48E-04 | -4.14E+08 | 9.38E-01 | 1.14E-01 | 1.88E-02 |
| 9 | 1.4 | 2.45E+05 | 1.25E-03 | 4.28E-04 | -3.78E+08 | 8.86E-01 | 2.24E-01 | 2.63E-02 |
| 9 | 1.5 | 3.16E+05 | 1.18E-03 | 5.38E-04 | -3.77E+08 | 8.63E-01 | 2.92E-01 | 2.40E-02 |
| 9 | 1.6 | 4.31E+05 | 9.52E-04 | 5.17E-04 | -3.56E+08 | 8.11E-01 | 4.15E-01 | 2.19E-02 |
| 9 | 1.7 | 4.84E+05 | 8.97E-04 | 4.43E-04 | -3.33E+08 | 7.77E-01 | 4.96E-01 | 3.00E-02 |
| 9 | 1.8 | 6.63E+05 | 7.00E-04 | 4.31E-04 | -3.03E+08 | 6.97E-01 | 6.82E-01 | 2.40E-02 |
| 9 | 1.9 | 7.63E+05 | 5.68E-04 | 3.70E-04 | -2.90E+08 | 6.67E-01 | 7.55E-01 | 2.46E-02 |
| 9 | 2 | 9.35E+05 | 5.08E-04 | 3.53E-04 | -2.72E+08 | 6.26E-01 | 8.63E-01 | 1.14E-02 |
| 10 | 1.1 | 1.45E+05 | 2.22E-03 | 4.54E-04 | -4.20E+08 | 9.88E-01 | 2.08E-02 | **5.74E-02** |
| 10 | 1.2 | 1.69E+05 | 1.92E-03 | 4.50E-04 | -4.20E+08 | 9.66E-01 | 6.10E-02 | 4.74E-02 |
| 10 | 1.3 | 1.97E+05 | 1.73E-03 | 5.23E-04 | -4.14E+08 | 9.28E-01 | 1.34E-01 | 2.00E-02 |
| 10 | 1.4 | 2.68E+05 | 1.36E-03 | 6.17E-04 | -4.01E+08 | 8.81E-01 | 2.33E-01 | 2.32E-02 |
| 10 | 1.5 | 2.68E+05 | 1.34E-03 | 4.09E-04 | -3.77E+08 | 8.68E-01 | 2.73E-01 | 3.10E-02 |
| 10 | 1.6 | 4.13E+05 | 1.03E-03 | 4.90E-04 | -3.50E+08 | 7.97E-01 | 4.33E-01 | 2.73E-02 |
| 10 | 1.7 | 5.16E+05 | 8.72E-04 | 4.30E-04 | -3.29E+08 | 7.60E-01 | 5.36E-01 | 3.88E-02 |
| 10 | 1.8 | 7.05E+05 | 7.55E-04 | 1.97E-04 | -3.15E+08 | 7.08E-01 | 6.75E-01 | 2.33E-02 |
| 10 | 1.9 | 8.76E+05 | 5.93E-04 | 3.57E-04 | -2.94E+08 | 6.73E-01 | 7.66E-01 | 2.21E-02 |
| 10 | 2 | 1.08E+06 | 4.38E-04 | 3.27E-04 | -2.69E+08 | 6.20E-01 | 8.97E-01 | 1.77E-02 |
| 11 | 1.1 | 1.40E+05 | 3.15E-03 | 5.52E-04 | -4.28E+08 | 9.86E-01 | 2.49E-02 | 2.06E-02 |
| 11 | 1.2 | 1.58E+05 | 2.56E-03 | 6.66E-04 | -4.32E+08 | 9.66E-01 | 6.25E-02 | 3.88E-02 |
| 11 | 1.3 | 1.65E+05 | 2.55E-03 | 4.65E-04 | -4.15E+08 | 9.45E-01 | 1.01E-01 | 2.06E-02 |
| 11 | 1.4 | 2.04E+05 | 1.71E-03 | 4.01E-04 | -4.03E+08 | 9.07E-01 | 1.81E-01 | 2.35E-02 |
| 11 | 1.5 | 2.65E+05 | 1.51E-03 | 4.01E-04 | -3.73E+08 | 8.61E-01 | 2.85E-01 | 2.55E-02 |
| 11 | 1.6 | 3.44E+05 | 1.27E-03 | 3.73E-04 | -3.57E+08 | 8.23E-01 | 3.80E-01 | 2.06E-02 |
| 11 | 1.7 | 5.16E+05 | 8.90E-04 | 4.06E-04 | -3.34E+08 | 7.65E-01 | 5.31E-01 | 1.34E-02 |
| 11 | 1.8 | 7.71E+05 | 6.77E-04 | 4.43E-04 | -3.19E+08 | 7.10E-01 | 6.78E-01 | 5.02E-03 |
| 11 | 1.9 | 9.28E+05 | 5.49E-04 | 5.16E-04 | -2.84E+08 | 6.58E-01 | 8.09E-01 | 1.21E-02 |
| 11 | 2 | 1.18E+06 | 4.62E-04 | 3.55E-04 | -2.74E+08 | 6.16E-01 | 9.26E-01 | 2.51E-02 |
| 12 | 1.1 | **1.38E+05** | **3.32E-03** | 4.57E-04 | -4.24E+08 | 9.87E-01 | 2.38E-02 | 1.73E-02 |
| 12 | 1.2 | 1.47E+05 | 3.17E-03 | 1.48E-03 | **-4.38E+08** | 9.66E-01 | 6.14E-02 | 8.74E-03 |
| 12 | 1.3 | 1.72E+05 | 2.17E-03 | 3.79E-04 | -4.15E+08 | 9.30E-01 | 1.28E-01 | 2.55E-02 |
| 12 | 1.4 | 1.87E+05 | 2.25E-03 | 3.70E-04 | -3.96E+08 | 9.16E-01 | 1.69E-01 | 2.06E-02 |
| 12 | 1.5 | 3.25E+05 | 1.31E-03 | 4.37E-04 | -3.68E+08 | 8.34E-01 | 3.40E-01 | 2.31E-02 |
| 12 | 1.6 | 3.50E+05 | 1.22E-03 | 3.40E-04 | -3.53E+08 | 8.24E-01 | 3.85E-01 | 3.95E-02 |
| 12 | 1.7 | 5.59E+05 | 8.73E-04 | 3.58E-04 | -3.38E+08 | 7.54E-01 | 5.53E-01 | 2.00E-02 |
| 12 | 1.8 | 6.33E+05 | 7.73E-04 | 2.80E-04 | -3.21E+08 | 7.45E-01 | 6.02E-01 | 3.88E-02 |
| 12 | 1.9 | 8.46E+05 | 5.70E-04 | 2.87E-04 | -2.96E+08 | 6.77E-01 | 7.66E-01 | 8.11E-03 |
| 12 | 2 | 1.03E+06 | 4.65E-04 | 2.53E-04 | -2.78E+08 | 6.38E-01 | 8.75E-01 | 2.16E-02 |
| 13 | 1.1 | 1.45E+05 | 2.32E-03 | 8.38E-04 | -4.31E+08 | 9.87E-01 | 2.42E-02 | 3.53E-02 |
| 13 | 1.2 | 1.49E+05 | **5.11E-03** | 3.73E-04 | -4.19E+08 | 9.69E-01 | 5.87E-02 | 3.72E-02 |
| 13 | 1.3 | 1.66E+05 | 2.96E-03 | 8.72E-04 | -4.32E+08 | 9.35E-01 | 1.16E-01 | 2.06E-02 |
| 13 | 1.4 | 2.28E+05 | 2.13E-03 | 3.89E-04 | -3.92E+08 | 9.08E-01 | 1.86E-01 | 2.30E-02 |
| 13 | 1.5 | 2.57E+05 | 1.56E-03 | 4.06E-04 | -3.99E+08 | 8.80E-01 | 2.59E-01 | 1.80E-02 |
| 13 | 1.6 | 3.57E+05 | 1.33E-03 | 3.82E-04 | -3.62E+08 | 8.26E-01 | 3.83E-01 | 2.09E-02 |
| 13 | 1.7 | 4.60E+05 | 1.03E-03 | 2.67E-04 | -3.40E+08 | 7.88E-01 | 4.82E-01 | 3.95E-02 |
| 13 | 1.8 | 8.23E+05 | 6.31E-04 | 6.28E-04 | -3.20E+08 | 7.10E-01 | 6.96E-01 | 1.99E-02 |
| 13 | 1.9 | 8.00E+05 | 6.21E-04 | 2.20E-04 | -3.01E+08 | 7.01E-01 | 7.19E-01 | 7.50E-03 |
| 13 | 2 | 1.00E+06 | 4.93E-04 | 1.99E-04 | -2.82E+08 | 6.60E-01 | 8.35E-01 | 2.20E-02 |
| 14 | 1.1 | **1.34E+05** | 2.92E-03 | 5.22E-04 | **-4.35E+08** | 9.89E-01 | 1.90E-02 | 4.10E-02 |
| 14 | 1.2 | 1.44E+05 | 2.30E-03 | 7.93E-04 | -4.26E+08 | 9.68E-01 | 6.07E-02 | 5.09E-02 |
| 14 | 1.3 | 1.55E+05 | 2.84E-03 | 3.02E-04 | -4.14E+08 | 9.45E-01 | 1.04E-01 | 2.30E-02 |
| 14 | 1.4 | 2.06E+05 | 2.28E-03 | 6.18E-04 | -3.92E+08 | 9.02E-01 | 1.92E-01 | 8.03E-03 |
| 14 | 1.5 | 2.55E+05 | 1.80E-03 | 7.70E-04 | -3.84E+08 | 8.79E-01 | 2.58E-01 | 1.80E-02 |
| 14 | 1.6 | 4.03E+05 | 1.25E-03 | 4.28E-04 | -3.63E+08 | 8.18E-01 | 4.02E-01 | 2.09E-02 |
| 14 | 1.7 | 5.60E+05 | 9.12E-04 | 5.69E-04 | -3.50E+08 | 7.88E-01 | 4.96E-01 | 3.83E-02 |
| 14 | 1.8 | 7.20E+05 | 7.56E-04 | 4.55E-04 | -3.28E+08 | 7.48E-01 | 6.10E-01 | 2.81E-02 |
| 14 | 1.9 | 9.47E+05 | 5.58E-04 | 2.47E-03 | -2.98E+08 | 6.71E-01 | 8.04E-01 | 2.09E-02 |
| 14 | 2 | 1.04E+06 | 4.97E-04 | 2.21E-04 | -2.78E+08 | 6.60E-01 | 8.46E-01 | 2.24E-02 |
| 15 | 1.1 | 1.46E+05 | 2.78E-03 | 8.37E-04 | -4.24E+08 | 9.83E-01 | 2.97E-02 | 3.26E-02 |
| 15 | 1.2 | 1.47E+05 | 3.25E-03 | 1.10E-03 | -4.25E+08 | 9.66E-01 | 6.25E-02 | 1.77E-02 |
| 15 | 1.3 | 1.60E+05 | 2.66E-03 | 3.39E-04 | -4.12E+08 | 9.35E-01 | 1.20E-01 | 2.30E-02 |
| 15 | 1.4 | 2.21E+05 | 2.39E-03 | 4.67E-04 | -3.99E+08 | 9.01E-01 | 1.95E-01 | 2.09E-02 |
| 15 | 1.5 | 2.45E+05 | 1.92E-03 | 2.67E-04 | -3.82E+08 | 8.73E-01 | 2.64E-01 | 2.68E-02 |
| 15 | 1.6 | 3.71E+05 | 1.37E-03 | 4.76E-04 | -3.68E+08 | 8.34E-01 | 3.70E-01 | 1.80E-02 |
| 15 | 1.7 | 5.02E+05 | 1.09E-03 | 2.71E-04 | -3.53E+08 | 7.84E-01 | 5.02E-01 | 2.30E-02 |
| 15 | 1.8 | 6.36E+05 | 8.61E-04 | 2.33E-04 | -3.32E+08 | 7.55E-01 | 5.94E-01 | 1.80E-02 |
| 15 | 1.9 | 8.86E+05 | 6.17E-04 | 3.47E-04 | -3.09E+08 | 7.07E-01 | 7.27E-01 | 3.27E-02 |
| 15 | 2 | 1.42E+06 | 3.60E-04 | 1.36E-03 | -2.68E+08 | 6.06E-01 | 9.98E-01 | 2.45E-02 |
