# Supplementary material for: Frequent but asymmetric niche shifts in Bulbophyllum orchids support environmental and climatic instability in Madagascar over Quaternary time scales
Source: BMC Evol Biol. 2016 Jan 19;16:14. doi: 10.1186/s12862-016-0586-3 (PMC4717530; doi:10.1186/s12862-016-0586-3)
Supplement: Additional file 2: — Rankings of loadings for the first two principal components (PC1, PC2) of 604 Bulbophyllum clade C (plus outgroup) locality values of 20 environmental variables (altitude, bioclim 1–19). (DOC 40 kb) [file 12862_2016_586_MOESM2_ESM.doc]

**Additional file 2**

**Gamisch et al. “Frequent but asymmetric niche shifts in *Bulbophyllum* orchids support environmental and climatic instability in Madagascar over Quaternary time scales”**

**Table S2** Rankings of loadings for the first two principal components (PC1, PC2) of 604 *Bulbophyllum* clade C (plus outgroup) locality values of 20 environmental variables (altitude, bioclim 1–19).

| **PC1 loading** | **Code** | **Variable** | **PC2 loading** | **Code** | **Variable** |
| --- | --- | --- | --- | --- | --- |
| -0.294 | alt | Altitude | 0.352 | bio4 | Temperature Seasonality |
| 0.287 | bio6 | Min Temperature of Coldest Month | -0.350 | bio15 | Precipitation Seasonality |
| 0.287 | bio10 | Mean Temperature of Warmest Quarter | -0.340 | bio3 | Isothermality |
| 0.286 | bio8 | Mean Temperature of Wettest Quarter | 0.307 | bio14 | Precipitation of Driest Month |
| 0.281 | bio9 | Mean Temperature of Driest Quarter | 0.305 | bio17 | Precipitation of Driest Quarter |
| 0.268 | bio1 | Annual Mean Temperature | 0.289 | bio19 | Precipitation of Coldest Quarter |
| 0.255 | bio11 | Mean Temperature of Coldest Quarter | -0.256 | bio5 | Max Temperature of Warmest Month |
| 0.251 | bio12 | Annual Precipitation | -0.231 | bio11 | Mean Temperature of Coldest Quarter |
| 0.217 | bio5 | Max Temperature of Warmest Month | -0.226 | bio2 | Mean Diurnal Range |
| -0.216 | bio7 | Temperature Annual Range | -0.204 | bio13 | Precipitation of Wettest Month |
| 0.213 | bio19 | Precipitation of Coldest Quarter | -0.200 | bio1 | Annual Mean Temperature |
| 0.202 | bio17 | Precipitation of Driest Quarter | -0.158 | bio9 | Mean Temperature of Driest Quarter |
| -0.201 | bio2 | Mean Diurnal Range | -0.143 | bio16 | Precipitation of Wettest Quarter |
| 0.201 | bio14 | Precipitation of Driest Month | 0.134 | bio12 | Annual Precipitation |
| 0.180 | bio16 | Precipitation of Wettest Quarter | -0.108 | bio10 | Mean Temperature of Warmest Quarter |
| 0.167 | bio13 | Precipitation of Wettest Month | 0.107 | bio18 | Precipitation of Warmest Quarter |
| -0.165 | bio15 | Precipitation Seasonality | -0.103 | bio7 | Temperature Annual Range |
| 0.144 | bio18 | Precipitation of Warmest Quarter | -0.102 | bio8 | Mean Temperature of Wettest Quarter |
| -0.098 | bio4 | Temperature Seasonality | -0.086 | bio6 | Min Temperature of Coldest Month |
| -0.097 | bio3 | Isothermality | 0.001 | alt | Altitude |
